# Lifelong impact of extreme stress on the human brain: Holocaust survivors study

**DOI:** 10.1016/j.ynstr.2021.100318

**Published:** 2021-03-20

**Authors:** Monika Fňašková, Pavel Říha, Marek Preiss, Petr Bob, Markéta Nečasová, Eva Koriťáková, Ivan Rektor

**Affiliations:** a Central European Institute of Technology (CEITEC), Brain and Mind Research Program, Masaryk University, Brno, Czech Republic; b Institute of Biostatistics and Analyses, Faculty of Medicine, Masaryk University, Brno, Czech Republic; c First Department of Neurology, St. Anne's Hospital and School of Medicine, Masaryk University, Brno, Czech Republic; d University of New York in Prague, Czech Republic

**Keywords:** Holocaust survivors, MRI, Posttraumatic stress, Posttraumatic growth, Lifelong impact

## Abstract

**Background:**

We aimed to assess the lifelong impact of extreme stress on people who survived the Holocaust. We hypothesised that the impact of extreme trauma is detectable even after more than 70 years of an often complicated and stressful post-war life.

**Methods:**

Psychological testing was performed on 44 Holocaust survivors (HS; median age 81.5 years; 29 women; 26 HS were under the age of 12 years in 1945) and 31 control participants without a personal or family history of the Holocaust (control group (CG); median 80 years; 17 women). Magnetic resonance imaging (MRI) using the 3T Siemens Prisma scanner was performed on 29 HS (median 79 years; 18 women) and 21 CG participants (median 80 years; 11 women). The MRI-tested subgroup that had been younger than 12 years old in 1945 was composed of 20 HS (median 79 years; 17 women) and 21 CG (median 80 years; 11 women).

**Results:**

HS experienced significantly higher frequency of depression symptoms, posttraumatic stress symptoms, and posttraumatic growth, and lower levels of well-being. The MRI shows a lifelong neurobiological effect of extreme stress. The areas with reduced grey matter correspond to the map of the impact of stress on the brain structure: insula, anterior cingulate, ventromedial cortex including the subgenual cingulate/orbitofrontal cortex, temporal pole, prefrontal cortex, and angular gyrus. HS showed good adjustment to post-war life conditions.

Psychological growth may contribute to compensation for the psychological and neurobiological consequences of extreme stress.

The reduction of GM was significantly expressed also in the subgroup of participants who survived the Holocaust during their childhood.

**Conclusion:**

The lifelong psychological and neurobiological changes in people who survived extreme stress were identified more than 70 years after the Holocaust. Extreme stress in childhood and young adulthood has an irreversible lifelong impact on the brain.

## 1 Introduction

The Holocaust was the most traumatic man-made event in European history. In the former Czechoslovakia, the entire Jewish population suffered from this large-scale genocide, which lasted from 1938 to 1945. It started with social and professional exclusion, humiliation, and suppression of basic rights, followed by deportation to concentration camps, forced labour, and exposure to horrific atrocities, or by illegally hiding or joining partisan groups under constant threat of discovery and execution. All of the holocaust survivors (HS), independently of their age, experienced massive trauma and the post-war shock of having lost family members, including parents, children, and siblings, and the necessity of adjusting to new and difficult life circumstances.

The first studies of the effect of this extreme stress on the health of the HS noted the mental impact but focused on physical health (Helweg-Larsen et al., 1952). The term ‘concentration camp syndrome’ for symptoms including emotional instability, poor concentration, and fatigue was introduced in the 1960s (Eitinger, 1962).

Levav and Abramson (Levav and Abramson, 1984) showed that 30 years after the war emotional distress had a higher prevalence in the former concentration camp inmates than in other European-born members of the community. Barel et al. performed a meta-analysis of 71 studies with 12,746 participants elucidating the long-term psychiatric, psychosocial, and physical consequences of the Holocaust. They found higher-level posttraumatic stress symptoms in HS but also adaptation (cognitive function, physical health, etc.) combining psychological growth with defense mechanisms (Barel et al., 2010). They marked this combination of chronic stress symptoms and resilience as ‘characteristics of the symptoms of Holocaust survivors’.

Traumatic stress is manifested by changes in brain structure. The hippocampus, amygdala, cingulate, prefrontal cortex (Arnsten et al., 2015; Bremner, 2003), and insular cortex (Paulus and Stein, 2006) are often mentioned as structures that are vulnerable to the effects of stress (Ansell et al., 2012; Bruce et al., 2013; Cohen et al., 2006; Kasai et al., 2008; Kuo et al., 2012; Lupien and Lepage, 2001; McEwen et al., 2016; McEwen and Morrison, 2013; Paulus and Stein, 2006; Roozendaal et al., 2009; Yaribeygi et al., 2017). Neurobiological modifications caused by stress can be linked with the development of diseases like depression (Bremner et al., 2000) and posttraumatic stress disorder (PTSD) (Bremner et al., 1995; Logue et al., 2018; Villarreal et al., 2002). Increased vulnerability to PTSD was observed in Holocaust survivors (Yehuda et al., 1998). In this study, we explored the lifelong impact of stress on brain structure using structural MRI.

The timing of stress exposure is a critical factor for the impacts of stress on brain structure and functions. A younger age during the traumatic period is linked to greater damage to personality development (Keilson and Sarphatie, 1992). During development (prenatal period, childhood, adolescence) and age-dependent changes (ageing), the brain is more vulnerable to the effects of stress hormones (Lupien et al., 2009). Childhood adversities are typically associated with dysregulation of the hypothalamic-pituitary-adrenal (HPA) axis and early childhood trauma can cause long lasting neurobiological and psychological deficiencies (Dye, 2018).

To explore the impact of stress during development, we investigated a subgroup of HS who were under 12 years old at the end of the war in 1945. Dividing the research set at the age of 12 makes sense from a developmental point of view; according to Erikson and Erikson, the fourth stage of human life ends at the age of 12. This is the last stage before adolescence (Erikson and Erikson, 1998).

The goal of this study is to assess the lifelong psychological and neurobiological impact of long-lasting extreme stress. The combination of psychological testing with brain MRI more than 70 years after the war provides unique data about the impact of extreme trauma.

Given the ages of survivors, this data probably reflects the last chance to explore the lifelong impact of this extreme trauma and directly learn from the survivors about their evaluations of a life marked by extreme stress. Data from the genetic part of this study have been partially published, including results about telomere length and mitochondrial DNA (Cai et al., 2020; Konečná et al., 2019).

**The study is based on two hypotheses:** 1. We hypothesised that Holocaust survivors have had a lifelong impact on stress-related brain areas combined with psychological consequences of stress as well as signs of posttraumatic growth, identifiable despite the complicated and often stressful life in Central Europe after the war.

2. We hypothesised that the lifelong consequences of extreme stress trauma would also be expressed in people who survived the Holocaust as children, despite the fact that children have a limited ability to cognitively process life-threatening situations (Sigal and Weinfeld, 2001) and the children were not exposed to direct threats of being killed, as most of them survived the Holocaust hidden in other families or institutions.

## 2 Methods

### 2.1 Participants and recruitment

#### 2.1.1 Research and recruitment

The study was conducted at the Central European Institute of Technology (CEITEC) Neuroscience Centre, at Masaryk University in Brno between 2015 and 2020. Part of the data was obtained at the National Institute of Mental Health (NÚDZ) in Klecany. The data were obtained according to the Declaration of Helsinki. The research was approved by the ethics committee at Masaryk University; informed consent was obtained from all participants.

All of the participants were of Czech or Slovak origin, i.e. people with a similar geopolitical background. The two countries formed one state – Czechoslovakia – until 1993, and the close connections between the citizens continue; the Czech and Slovak languages are mutually comprehensible.

The participants were recruited through the cooperation of local Jewish communities (the Holocaust survivors group), announcements in the media, and postings on the university website. For HS and CG recruitment, we also used personal invitations from members of the research team and the snowball sampling method. The CG was completed when the composition of HS was already clear and the CG could be matched with HS.

#### 2.1.2 Participants characteristics

Exclusion criteria: a history of treatment for severe psychiatric disorders (such as psychosis), any kind of severe brain impairment (brain injury, tumours, neurodegenerative diseases), and significant cognitive decline (all participants scored over 26 points in the Mini-Mental State Examination) (Solomon et al., 1998). Contraindications for MRI were metal implants, pacemakers, and claustrophobia.

The HS and CG groups were not significantly different in age, sex, and education; this was verified using Mann-Whitney *U* test. The groups were 44 HS with median age 82 (71–95) years, 29 women (66%) and 31 Czech and Slovak non-Jewish control participants not exposed to war-related trauma with median age 80 (73–90) years, 17 women (55%). Higher education had been attained by 46% of HS and 36% of CG.

The subgroup under 12 years old in 1945 was composed of 26 HS with median age 78.5 (71–84) years, 17 women (65%) and 24 control participants with median age 78 (73–84), 12 women (50%). Table with demographic group characteristics is in the supplementary material.

Participants who could not participate in MR scanning because of contraindications or who underwent MR scanning with insufficiently quality scans were excluded from the final brain images analysis.

The neuroimaging cohort was composed of 29 HS with median age 79 (72–95), 18 women (62%), and 21 control participants median age 80 (73–86), 11 women (52%). Their psychological profile resembled the profile of the whole cohort (Table 2).

The neuroimaging subgroup under 12 years old in 1945 was composed of 20 HS with median age 78 (72–84), 12 women (60%), and 21 control participants median age 80 (73–86), 11 women (52%).

No gender-associated effects were found using a two-sample *t*-test comparing male and female data.

#### 2.1.3 Background of examined groups

##### 2.1.3.1 Holocaust survivors group characteristics

During the Holocaust, 24 HS were in hiding, e.g. living with a non-Jewish family in a small village, in a farmhouse, in an evangelist orphanage, or in a secret room, in their childhood or adolescence. Five HS lived under a false identity or were hiding in the mountains; some of them joined the partisan army.

Fifteen HS were imprisoned in a ghetto (most often Terezín - Theresienstadt) and/or in concentration camps (Auschwitz, Bergen-Belsen, Buchenwald, Dachau, or Mauthausen). Several participants had survived a death march. The persecution increased over six years; the immediate danger of execution, whether after being discovered for people who were hiding or after being imprisoned in a concentration camp, lasted from six months to four years.

Most of them experienced trauma at critical developmental phases; 26 HS were aged under 12 years by the end of the war in 1945. Nineteen of them were in hiding (nine with their parents and ten without them). Seven were imprisoned in the ghetto Theresienstadt.

##### 2.1.3.2 Control group characteristics

The Czech Republic was occupied by Nazi Germany as the Protektorat Böhmen und Mähren. There was a strong oppression of the Czech population, but its majority was not directly exposed to the war events. Participants in CG were civilians and did not participate in military action or resistance; during the war, they were not under direct life-threatening danger.

**Post-war life conditions** under the communist regime from 1948 to 1989 were difficult and differed from the conditions in Western democracies. The regime was oppressive and often anti-Semitic, in particular in the 1950s, with a series of political processes followed by executions and long-term imprisonments. Jews experienced direct oppression, as did other groups, e.g. private farmers and entrepreneurs, Christians, intellectuals, etc. After a liberalisation period in the 1960s, ended by the Soviet army intervention in 1968, a general suppression of human rights followed for 20 years. Based on individual interviews, we can state that none of our study participants advanced their career based on membership in the Communist Party. In principle, the HS and CG suffered from the communist oppression in a more or less similar way.

### 2.2 Initial screening

For the initial screening, all participants were tested with the 7-min screen test (Solomon et al., 1998).

The protocol consisted of interviews, psychological questionnaires, and MR scanning. In addition, participants completed the Geriatric Depression Scale test as a part of the initial screening (participants with major depression were not included in the study).

### 2.3 Interview

All participants, HS and CG, were asked about their life before, during, and after the war. HS were also asked how they survived the Holocaust and how long they were persecuted. In the self-report part, the HS answered a short questionnaire focused on the self-evaluation of their personal life and professional career as affected by the Holocaust.

### 2.4 Psychological measures

Four questionnaires testing the hypothesis of the lifelong impact of extreme stress were chosen for this study. Two tests explored the negative impact of stress, specifically posttraumatic stress disorder (PTSD) symptoms and actual stress symptoms (PCL-C and TSC-40 respectively); one test explored the positive impact of stress (PTGI); and one test explored the subjective appreciation of actual quality of life (SOS-10).

All psychological questionnaires are summarised in detail in Table 1.

**Table 1:** Psychological tests.

GDS	*The Geriatric Depression Scale*	The 15-item version of the Geriatric Depression Scale (GDS-15) was used. The GDS-15 has been employed in both practice and research across different groups of elderly people (Burke et al., 1991). A cut-off score of 6 points was used.
**PCL-C**	***PTSD Checklist – Civilian Version***	The PTSD Checklist – Civilian Version is a 17-item self-report measure of the DSM-IV symptoms of PTSD. The PCL-C is a screening instrument that asks respondents to consider a ‘list of problems and complaints that people sometimes have in response to stressful experiences’ and to indicate how much they ‘have been bothered by each problem in the past month’ on a scale of 1 = not at all to 5 = extremely. A higher score is associated with a greater level of PTSD symptoms (Conybeare et al., 2012). A cut-off score of 31 points was used.
**TSC-40**	***Trauma Symptom Checklist***	The Trauma Symptom Checklist – 40 is a self-report measure with 40 items scored on a 4-point Likert scale (From 0 = never to 3 = often; total score from 0 to 120; a higher score is interpreted as a higher level of traumatic stress). The TSC-40 measure includes subscales for dissociation, anxiety, depression, and sleep disturbances (Elliott and Briere, 1992).
**PTGI**	***The Post Traumatic Growth Inventory***	The 21-item Post Traumatic Growth Inventory was used to assess positive change as a result of the struggle with stressful experiences. Participants were asked to identify the degree to which they experienced a particular change (0 = I did not experience this change as a result of my crisis to 5 = I experienced this change to a very great degree). The score range for the total PTGI is 0–105, with higher scores indicative of greater growth (Steffens and Andrykowski, 2015; Tedeschi and Calhoun, 1996). A cut-off score of 46 points was used.
**SOS-10**	***Schwartz Outcome Scale-10***	The scale represents a broad construct related to multiple aspects of psychological functioning and psychological well-being. The10-item measure was rated by respondents on a 7-point scale ranging from 0 = never to 6 = all the time (Young et al., 2003). The total SOS scores ranged from 0 to 60. A higher score is associated with greater well-being (Dragomirecka et al., 2006). A cut-off score of 40 points was used.

**Table 2:** Psychological questionnaires: differences between Holocaust survivors (HS) and the control group (CG).

*Group*	TSC-40		PCL-C		PTGI		SOS-10	
	HS	CG	HS	CG	HS	CG	HS	CG
***Median***	15.5	8	30	22	60.5	40	48.5	54
***Mean***	16.5	7.9	32.7	22.7	59.9	36.5	46.5	52.4
***Min***	0	0	17	17	7	0	18	29
***Max***	54	26	70	33	95	87	60	60
***P***	***0.006***		***0.0001***		***0.0001***		***0.002***	

TSC: Trauma Symptom Checklist; PCLC – C: PTSD Checklist – Civilian Version; PTGI: Posttraumatic Growth Inventory; SOS – Schwartz Outcome Scale.

#### 2.4.1 Statistical analysis

The results of the psychological questionnaires were summarised using median, minimum, and maximum. For statistical testing of differences between HS and GC, a non-parametric Mann-Whitney *U* test was used. The significance level for all statistical tests was set to p < 0.05. The statistical analyses were performed using STATISTICA 12. The effect of age was tested by multiple regression.

### 2.5 MR imaging

#### 2.5.1 Data acquisition

MR examinations were performed on a 3T scanner Siemens Prisma using a 64-channel head coil. The MRI protocol for voxel-based morphometry included 3D T1-weighted magnetisation prepared rapid gradient echo (MPRAGE) sequence with TR = 2.3 s, TE = 2.33 ms, TI = 0.9 s, FA = 8°, isometric voxel size 1 mm in FOV 224 × 224 mm and 240 slices.

Part of the data was obtained at a partner workplace, NÚDZ Klecany, with the same type of 3T Prisma scanner, multichannel coil, and protocol sequence.

Data from all participants were manually checked for artifacts and pathology was checked by an experienced radiologist. Participants who did not meet our quality criteria (scans without technical artifacts or lower SNR; scans without significant movement artifacts; participants with brain pathology; and scans without successfully finished segmentation and normalization into MNI space) were excluded from the study.

#### 2.5.2 Data processing

Anatomical MRI data were analysed using SPM12 (www.fil.ion.ucl.ac.uk) and CAT12 toolbox (www.neuro.uni-jena.de/cat) running in Matlab R2017b.

Individual data were adjusted for spatial inhomogeneity with an intensity normalization filter and then denoised with the Non-Local Means (SANLM) denoising filter. High resolution data were then segmented into grey matter using the SPM Tissue Probability Map (TPM) and registered into common MNI space using shooting template IXI555_MNI152_GS. Finally, spatially normalised and modulated GM maps were smoothed with 6 mm FWHM isotropic Gaussian kernel.

#### 2.5.3 Statistical analysis

Group statistics for stress effects were calculated with a second-level model using SPM12. The modulated GM images were multiplicatively corrected with total intracranial volume and then analysed. A two-sample *t*-test comparison of GMV files between stress group (respectively stress subgroup under 12 years of age) and the control group was performed; sex, age, and MRI machine were included as nuisance variables.

Resultant t-statistic maps were initially thresholded at a P value of <0.005 uncorrected and then only significant clusters at P < 0.05 FWE cluster level were picked.

#### 2.5.4 Grey matter volume and PTSD checklist scale correlation

Based on the Automated Anatomical Labeling (AAL) atlas (Tzourio-Mazoyer et al., 2002), we selected the mean GM volume in an area consisting of the ACC, OFC, and insula, which are constantly repeated stress areas in the literature (Ansell et al., 2012; Bolsinger et al., 2018; Kasai et al., 2008). These GM volumes and PCL-C values were correlated using Pearson correlation in HS to examine the relationship between brain morphology and posttraumatic stress manifestation.

## 3 Results

### 3.1 Interview

In the interviews with the Holocaust survivors, respondents from the focal group typically cited war events (e.g. death of parents, war as a whole, hiding during the war, transport to and stay in a concentration camp, loss of a loved one), as well as topics related to communism (e.g. secret police interrogations, anti-Semitism) and health problems (e.g. ventricular fibrillation, partial disability, accident, illness) as dominant life events. The control group was typically dominated by lifetime losses (e.g. parents, spouse) and health problems (e.g. heart attack, broken arm).

#### 3.1.1 Self-report

All HS participants were asked how they evaluate their current life in relation to the Holocaust. The questions were as follows: 1. Was the Holocaust the worst experience of your life? (84.1% answered yes or rather yes); 2. Did the Holocaust have a lifelong negative influence on your life? (70.5% answered yes or rather yes); 3. Are you satisfied with your personal life (lifelong view)? (79.6% answered yes or rather yes); 4. Are you satisfied with your career (lifelong view)? (86.4% answered yes or rather yes).

### 3.2 Psychological measures

#### 3.2.1 Depression symptoms

Depression symptoms were screened using the GDS. The prevalence of depression symptoms was significantly higher in HS (p < 0.001): depression symptoms were experienced by 15 HS (34.1%) and by 3 participants of CG (9.7%).

#### 3.2.2 Psychological testing

The results of the psychological testing (Table 2) significantly differed between HS and CG in all questionnaires. PCL-C showed higher rates of lasting symptoms of chronic stress in HS (in 21; 47.7%) than in CG (2; 6.5%). PTGI presents a higher rate of posttraumatic growth in HS, in 31 (70.5%), than in CG, in 11 (35.5%). SOS-10 displays a lower rate of well-being in HS, who could be classified as ‘maladjusted’ (9; 20.5%) than in CG (3; 9.7%). The effect of age was not statistically significant when used as covariate.

The results of the psychological testing in a subgroup of participants who were under the age of 12 in 1945 (Table 3) significantly differed between HS and CG in all questionnaires. PCL-C showed higher rates of lasting symptoms of chronic stress in HS (in 13; 50%) than in CG (1; 4.2%). There is a higher rate of posttraumatic growth in HS, in 18 (69.2%) than in CG, in 9 (37.5%). SOS-10 displays a lower rate of well-being in HS (6; 23.1%) than in CG (1; 4.2%).

**Table 3:** Psychological questionnaires: differences between HS under age of 12 in 1945 and age-matched CG.

*Group*	TSC-40		PCL-C		PTGI		SOS-10	
	HS	CG	HS	CG	HS	CG	HS	CG
***Median***	12	5.5	30.5	21.5	63	40.5	50	54.5
***Mean***	15.7	7.4	32.5	22.3	59.8	36.8	46.6	53.5
***Min***	0	0	17	17	7	0	18	38
***Max***	45	26	70	31	94	87	60	60
***P***	***0.03***		***0.002***		***0.004***		***0.01***	

TSC: Trauma Symptom Checklist; PCLC – C: PTSD Checklist – Civilian Version; PTGI: Posttraumatic Growth Inventory; SOS – Schwartz Outcome Scale.

### 3.3 MRI

#### 3.3.1 Neuroimaging group characteristics

The neuroimaging cohort psychological profile resembled the profile of the whole cohort (Table 4). No gender-associated effects were found using a two-sample *t*-test comparing male and female data.

**Table 4:** Psychological questionnaire results of participants participating in the neuroimaging part of this study.

*Group*	TSC-40		PCL-C		PTGI		SOS-10	
	HS	CG	HS	CG	HS	CG	HS	CG
***Median***	14	8	30	22	59	44	48	55
***Mean***	14.7	8.6	31.3	22.8	56.6	42.8	46.9	53.2
***Min***	0	1	19	17	7	1	18	38
***Max***	35	26	54	33	87	87	60	60
***P***	***0.009***		***0.002***		***0.05***		***0.006***	

Psychological test results are similar to those in the entire HS group: a greater rate of chronic stress symptoms (significantly in PCL-C) and a lower rate of well-being in HS. Posttraumatic growth is stronger in the HS group but with a borderline p-value of 0.0504.

#### 3.3.2 GMV reduction in holocaust survivors

**Fig. 1:**
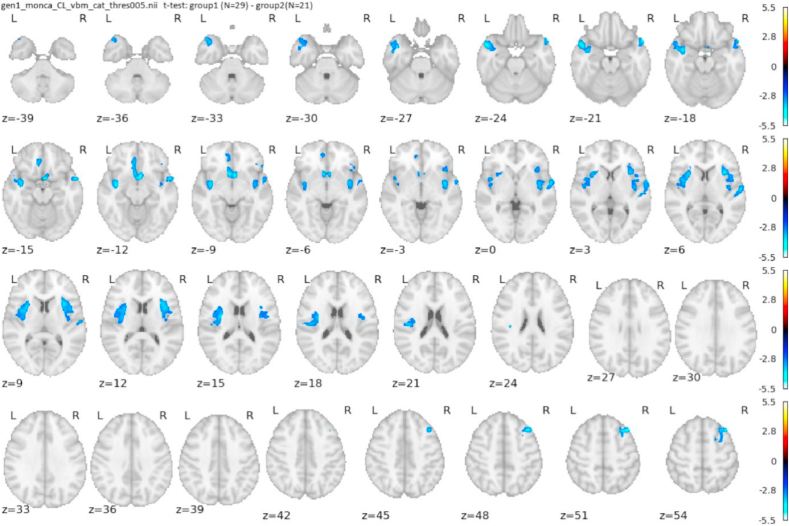
Structural MRI. Holocaust survivors vs control participants thresholded at 0.005; axial slices.

VBM showed a significant GM volume reduction in HS in regions described in Table 5 and Fig. 1.

**Table 5:** Holocaust survivors vs control group: Structural MRI, clusters with significant GM reduction compared control group. Initial threshold 0.005 uncorrected, 0.05 FWE cluster level significance.

Laterality	Structure	p-corrected	cluster size [cm3]	Coordinates	T-values
R	**Insula**	0.0079	7.31	47; −8; 18 41; 5; −6 53; 29; −11	−2.6897 −2.6898 −2.6905
	**Dorsolateral prefrontal cortex**	0.0100	3.64	39; 22; 52	−2.6906
	**Temporal pole, superior temporal gyrus**	0.0385	3.32	54; 6; −21	−2.6907
R and L	**BA 25 - subgenual cingulate**	0.0143	3.15	1; 6; −14	−2.6901
	**Anterior cingulate**				
L	**Temporal pole, superior temporal gyrus, insula**	0.0004	12.35	−35; 0; −17 −54; 5; −17 −42; −6; 2	−2.6896 −2.6899 −2.6901

Significant results up to p = 0.05 FWE cluster level. Larger clusters overlap several structures and can be divided into substructures for interpretation purposes. R – right, L – left, R and L – cluster covering bilateral medial cortices. Coordinates indicate the location with the maximum cluster value.

#### 3.3.3 GMV reduction in holocaust survivors under 12 years in 1945

**Fig. 2:**
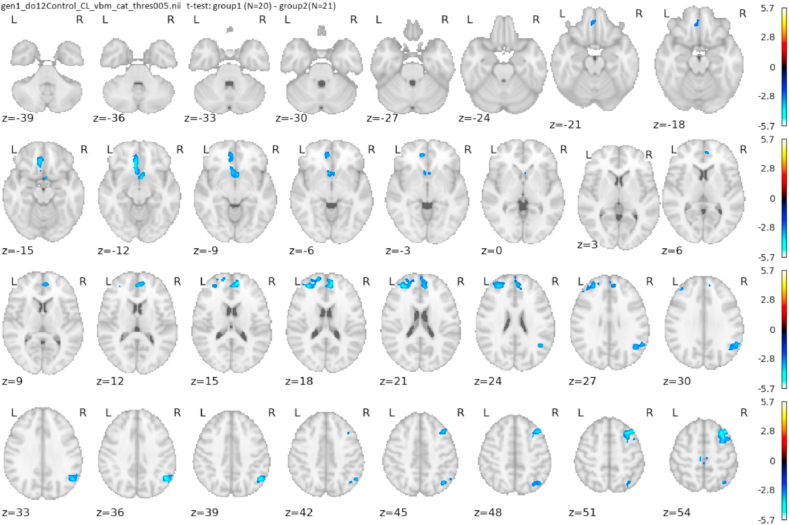
Structural MRI. Holocaust survivors younger than 12 years in 1945 vs control participants, thresholded at 0.005; axial slices.

VBM showed a significant GM volume reduction in HS under 12 years in regions described in Table 6 and Fig. 2.

**Table 6:** Holocaust survivors, age under 12 years in 1945. Structural MRI, clusters with significant GM reduction compared to control group. Initial threshold 0.005 uncorrected, 0.05 FWE cluster level significance.

Laterality	Structure	p-corrected	cluster size [cm3]	Coordinates	T-values
R	**Dorsolateral prefrontal cortex**	0.0030	5.10	39; 23; 54	−2.7195
	**Angular gyrus**	0.0384	3.22	48; −59; 38	−2.7242
R and L	**BA6 – medial precentral cortex**	0.0053	3.90	−5; −32; 71	−2.7206
	**Medial prefrontal cortex, anterior cingulate**	0.0251	2.30	8; 44; 15	−2.7275
L	**BA 25 – subgenual cingulate, orbitofrontal cortex**	0.0155	3.43	−9; 26; −11	−2.7199
	**BA 10, superior frontal gyrus**	0.0320	2.87	−17; 57; 21	−2.7022

Significant results up to p = 0.05 FWE cluster level. Larger clusters overlap several structures and can be divided into substructures for interpretation purposes. R – right, L – left, R and L – cluster covering bilateral medial cortices. Coordinates indicate the location with the maximum cluster.

Structural MRI map of Holocaust survivors younger than 12 years in 1945 vs control participants with a liberal initial threshold of p = 0.01 is presented in Supplementary Material. The comparison of the subgroups of HS under 12 years did not yield a qualitatively different pattern from the comparison of the entire groups of participants when lowering the threshold to compensate for the small group sizes.

#### 3.3.4 Correlation between GMV and PTSD symptoms

We found a significant correlation r = 0.395, p-value = 0.034, between grey matter volume in the stress-related network comprising ACC, OFC, and the insula and PCL-C test score.

## 4 Discussion

Extreme stress in childhood and young adulthood has an irreversible lifelong impact on the brain. More than 70 years after World War II, it is possible to identify lifelong psychological and neurobiological changes in people who survived the Holocaust as compared to a control group without a similar trauma history. There are apparent persistent differences in the frequency of depression symptoms, posttraumatic stress symptoms, and posttraumatic growth, in levels of well-being, and in GM volume in the brain.

Voxel-based morphometry (VBM) displayed a significant GM volume reduction in the HS as compared to CG. The areas of reduced grey matter correspond to the map of the impact of stress on the brain structure: insula, anterior cingulate, ventromedial cortex including the subgenual cingulate/orbitofrontal cortex, temporal pole, prefrontal cortex, and angular gyrus. The reduced structures were reported in connection with stress, emotions, affective disorders, autobiographical memory cognition, and behaviour.

The massive reduction of insular volume is of particular note. The insula is functionally linked with other structures that showed volume reduction in HS, in particular with anterior cingulate (ACC), ventromedial prefrontal, and orbitofrontal cortex (OFC) (Perez et al., 2017; Phillips et al., 2003). The anterior insula may be critical for processing emotions, self-awareness (Stevens and Jovanovic, 2019), and in disorders of mood and anxiety (Rolls et al., 2018).

The ACC is a limbic region associated with a multitude of cognitive and affective processes (Perez et al., 2017) including fear regulation Diekhof et al. (2011) (Drevets et al., 2008); and social behaviour (Devinsky et al., 1995). The medial prefrontal cortex includes the pregenual/subcallosal ACC, subgenual cingulate, and OFC and is associated with the processing of emotions, emotional behaviour, and memory (Noriuchi et al., 2019). The subgenual cingulate (BA 25) is being used as a target for deep brain stimulation therapy for major depression (Rolls et al., 2018).

The temporal pole (TP) is a paralimbic region involved in the regulation of emotion (Holland et al., 2011). A GM reduction in the left medial temporal gyrus and right superior frontal gyrus, possibly associated with autobiographical memory retrieval, was described in PTSD (Li et al., 2014). The angular gyrus is linked to several cognitive functions including self-referential processing (Stevens and Jovanovic, 2019). In a combat veteran PTSD study, the burden of psychological trauma across the lifespan correlated with reduced cortical thickness in limbic/paralimbic areas and in the medial precentral and dorsolateral prefrontal cortices (Lindemer et al., 2013).

It can be summarised that the regions with reduced GM volume are associated with functions that could have been influenced by extreme stress. Sustained stress exposure leads to persistent changes in brain circuits regulating behaviour and emotion (Arnsten et al., 2015). This appears even more evident when looking at these regions from the network perspective. The insula is a core region of the *salience network* that is involved in dynamic prioritising of internal and external stimuli and is implicated in mood/anxiety disorders (Perez et al., 2017). The reduced volume of the insula, ACC, and OFC is considered a sign of increased vulnerability to stress (Bolsinger et al., 2018). Cumulative lifetime adverse events were associated with reduced insular, subgenual ACC, and medial prefrontal volumes (Ansell et al., 2012). The regulation of emotions and of self-awareness are processed in a network composed of the insula and perigenual ACC/ventromedial prefrontal cortex (Perez et al., 2015). The map of reduced GM volume in HS is nearly identical with the set of regions involved in social cognition (Stevens and Jovanovic, 2019).

The affected regions belong to the three core neurocognitive systems crucial for cognitive and affective processing: the salience network, the default mode network, and the central executive network. Deficits in the three networks are associated with a wide range of stress-related psychiatric disorders such as anxiety, depression, and posttraumatic stress disorder (Menon, 2011).

Extreme trauma experienced in childhood has demonstrably lifelong consequences. The reduction of GM was significantly expressed in the young HS, who were under the age of 12 years in 1945. The brains of children are vulnerable despite the fact that children have a limited ability to cognitively process life-threatening situations (Sigal and Weinfeld, 2001). The GM volume reduction in children is probably a consequence of maladaptive experience-dependent neuroplastic changes that are more expressed in a developing brain (Thomason and Marusak, 2017). A lower GM volume in the ACC was found in individuals with prenatal stress (Marečková et al., 2019). Early-life adverse events have been associated with smaller insula, ACC, and OFC (Dannlowski et al., 2012; Rolls et al., 2018).

There were no observable changes in the hippocampus and amygdala. The volume reduction of the two structures has been reported in PTSD and affective disorders (Bremner, 2006, 2007; Teicher et al., 2003) but findings are not consistent. Earlier studies also did not find a reduction of the two structures in HS with PTSD (Cohen et al., 2006; Golier et al., 2005).

Several hypotheses explain the mechanisms of the alterations in brain structure induced by stress. Activation of the hypothalamic-pituitary-adrenal axis leads the increased release of corticosteroids which can exert a negative effect on neurogenesis and an increase in apoptosis (Li et al., 2014). However, a decrease in GM volume associated with a reduction in glia, with no loss of neurons, was described in ACC (Drevets et al., 2008). In a stress model in mice, the GM reduction was explained by the loss of dendrites (Blais et al., 1999; Kassem et al., 2013).

The GM reduction in our study is very probably the consequence of major psychological trauma. It is not explained by the effects of malnutrition on the brains of the survivors, as the majority of surviving children (with significant GM reduction) were hidden in non-Jewish families and did not experience extreme malnutrition. We found a significant correlation between grey matter volume in structures forming the stress network (insula, ACC, OFC) and PCL-C test score. This means that there is a clear link in our data between the grey matter volume and the psychological manifestations of posttraumatic stress symptoms.

To summarise the MRI part of our study: it shows an enduring lifelong effect of extremely stressful trauma on brain structure. The GM reduced areas correspond to the map of the impact of stress on the brain. The published studies mostly report the impact of stress on the human brain after a limited time period and do not address the question of whether the structural changes are reversible. Our data showing the lifelong consequences more than 70 years after extreme stress indicate that the GM reduction is irreversible. On the other hand, it is evident that the consequences of extreme stress can be compensated on a psychological level.

The psychological testing and HS interviews confirmed the profile corresponding to this structural map; however, the life course and other psychological signs display a more complicated and more positive pattern. After World War II, the psychopathology that characterised Holocaust survivors were described as a combination of chronic anxiety, depression, feelings of guilt, emotional instability, memory disturbances, and personality problems, alongside unresolved mourning and sadness (Barel et al., 2010; Chodoff, 1963; Graaf, 1975; Helweg-Larsen et al., 1952; Prager and Solomon, 1995; Sagi-Schwartz et al., 2003).

In our study, the HS, when compared to CG, presented a more frequent occurrence of symptoms of chronic stress and depression and lower levels of well-being scores. On the other hand, the HS presented signs of resilience that probably considerably influenced their post-war life (Heitlinger, 2011). They presented higher posttraumatic growth than the CG, and their self-estimation of their lives over the more than 70 years since the Holocaust showed a surprisingly positive pattern. The HS declared that they were satisfied with their lifelong personal life (in 79.6%) and with their professional careers (86.4%). That means that most of HS had productive and successful lives despite the atrocities they endured.

Surviving the Holocaust led to different reactions, including frequent suicides after the war. Those who were available for investigations for several decades after the Holocaust showed successful adaption capacities, similar to our study. The meta-analysis by Barel et al. elucidating the long-term consequences of the Holocaust for survivors suggested that alongside profound sadness there is room for growth (Barel et al., 2010). Several studies have provided support for resilience in survivors of other genocides and persecutions, such as in Bosnia and Cambodia (Ferren, 1999; Rousseau et al., 2003).

Holocaust survivors are not a homogeneous group and they vary in their post-trauma adjustment. Our study surpasses other published studies in the time that elapsed since the Holocaust – 70 to 75 years. The HS were up to 95 years old. We can speculate that surviving the Holocaust and living to a very advanced age could reflect a personality profile. It has been shown that Polish Holocaust survivors who immigrated to the British Mandate for Palestine after 1945 lived longer than the Polish Jews who immigrated before 1939, i.e. before the Holocaust (Sagi-Schwartz et al., 2013). The results of a study of Holocaust survivors aged 75 and older revealed almost no differences regarding the sociodemographic and interpersonal variables when compared to a control group. Nevertheless, survivors were found to be more vulnerable (Landau and Litwin, 2000).

Based on our data, we suggest that the combination of depression and chronic stress symptoms with GM reduction in critical areas and posttraumatic growth with good adaptation to life present characteristics of Holocaust survivors. It appears that the strong motivation of Holocaust survivors to rebuild their lives manifested itself primarily in raising families, becoming involved in social activities, and showing achievements on a wide spectrum of social functioning (Joffe et al., 2003; Krell, 1993). The neurobiological consequence of extreme stress, i.e. reduction of GM in areas related to stress symptoms, may be compensated by resilience and psychological growth. The lifelong consequences of the Holocaust on survivors may help to understand the adaptational challenges for survivors of more recent wars and catastrophic events.

**A brief conclusion** of our study is that Holocaust survivors continue to show neurobiological and psychological signs of having been traumatised even more than 70 years after the extreme stress. Extreme stress in childhood and young adulthood has an irreversible lifelong impact on the brain.

## 5 Limitations

• The fact that the study was conducting with older participants limited the time available for testing. A selection of brief psychological tests was chosen. The investigation lasted from 3.5 to 5 h. Participants were evaluated for depression symptoms but emotions were not otherwise tested; they were partially revealed in the interview.
• We did not detect lifetime symptom stresses. The gold standard for posttraumatic stress disorder (CAPS; Clinician-Administered PTSD Scale for DSM-5) was not used, as it is time consuming.
• The old age of the participants also limited the number of participants with MR data in sufficient quality.
• The control group was composed of people with no Jewish heritage. In Central Europe, it is not possible to find Jewish participants who were not affected by the Holocaust. Otherwise, the geopolitical background of all participants was similar.

## CRediT authorship contribution statement

**Monika Fňašková:** Project administration, Investigation, Resources, Data curation, Writing – original draft, Visualization. **Pavel Říha:** Methodology, Software, Formal analysis, Investigation. **Marek Preiss:** Conceptualization, Methodology, Validation. **Petr Bob:** Conceptualization, Methodology, Validation. **Markéta Nečasová:** Investigation, Formal analysis. **Eva Koriťáková:** Formal analysis. **Ivan Rektor:** Conceptualization, Supervision, Writing – original draft.

## Declaration of competing interest

None.

## Data Availability

All data are available upon request at the Repository CEITEC Masaryk University, MAFIL CF. The authors assert that all procedures contributing to this work comply with the ethical standards of the relevant national and institutional committees on human experimentation and with the Helsinki Declaration of 1975, as revised in 2008.
