# Supplementary material for: Lifelong impact of extreme stress on the human brain: Holocaust survivors study
Source: Neurobiol Stress. 2021 Mar 20;14:100318. doi: 10.1016/j.ynstr.2021.100318 (PMC8039853; doi:10.1016/j.ynstr.2021.100318)

*Figure: Structural MRI. Holocaust survivors younger than 12 years in 1945 vs control participants, thresholded at 0.01; axial slices*


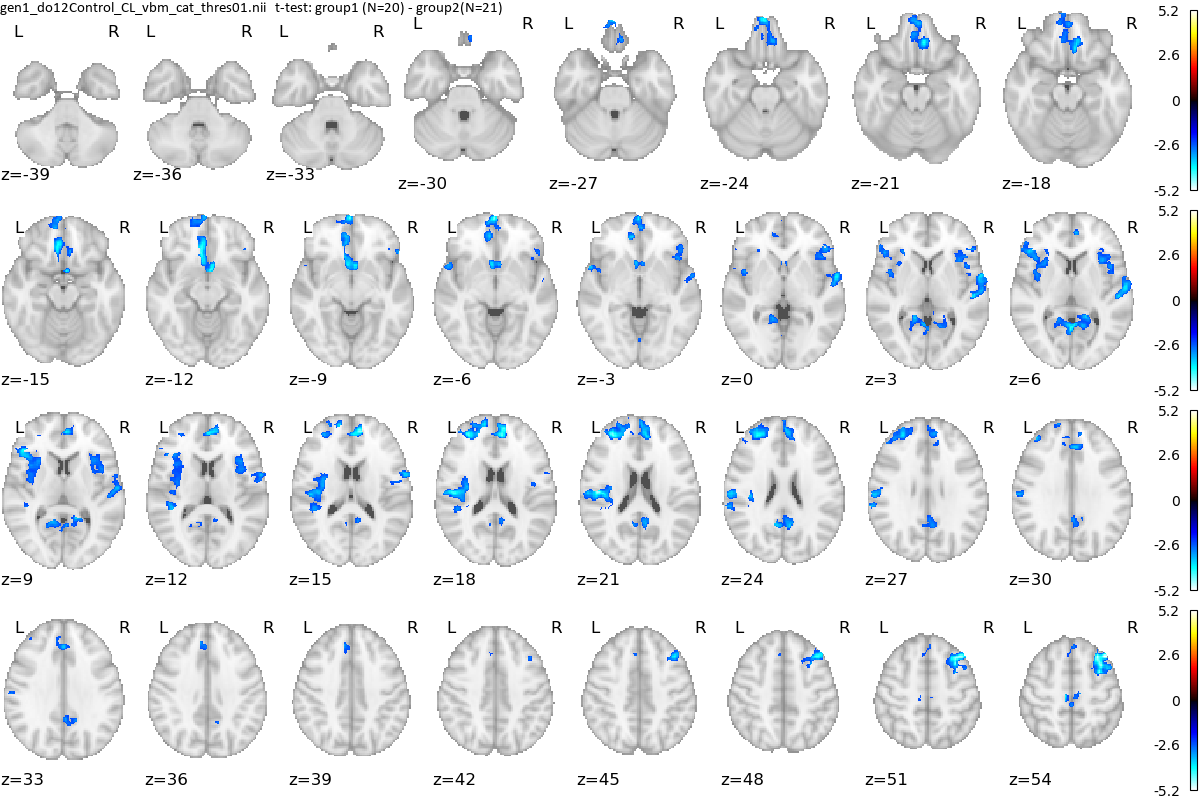

Supplement: Multimedia component 2 [file mmc2.docx]
